# RECQL1 and WRN DNA repair helicases: potential therapeutic targets and proliferative markers against cancers

**DOI:** 10.3389/fgene.2014.00441

**Published:** 2015-01-09

**Authors:** Kazunobu Futami, Yasuhiro Furuichi

**Affiliations:** GeneCare Research Institute Co., Ltd.Kamakura, Japan

**Keywords:** RecQ helicases, genome instability, mitotic catastrophe, RECQL1-siRNA, WRN-siRNA, anticancer drug candidates, chemotherapy, DDS

## Abstract

RECQL1 and WRN helicases in the human RecQ helicase family participate in maintaining genome stability, DNA repair, replication, and recombination pathways in the cell cycle. They are expressed highly in rapidly proliferating cells and tumor cells, suggesting that they have important roles in the replication of a genome. Although mice deficient in these helicases are indistinguishable from wild-type mice, their embryonic fibroblasts are sensitive to DNA damage. In tumor cells, silencing the expression of RECQL1 or WRN helicase by RNA interference induces mitotic catastrophe that eventually kills tumor cells at the mitosis stage of the cell cycle. By contrast, the same gene silencing by cognate small RNA (siRNA) never kills normal cells, although cell growth is slightly delayed. These findings indicate that RECQL1 and WRN helicases are ideal molecular targets for cancer therapy. The molecular mechanisms underlying these events has been studied extensively, which may help development of anticancer drugs free from adverse effects by targeting DNA repair helicases RECQL1 and WRN. As expected, the anticancer activity of conventional genotoxic drugs is significantly augmented by combined treatment with RECQL1- or WRN-siRNAs that prevents DNA repair in cancer cells. In this review, we focus on studies that clarified the mechanisms that lead to the specific killing of cancer cells and introduce efforts to develop anticancer RecQ-siRNA drugs free from adverse effects.

## INTRODUCTION

The RecQ helicase family is conserved in all organisms and participates in maintaining the genomic integrity of cells. Microorganisms, such as *Escherichia coli* and *Saccharomyces cerevisiae*, contain only one RecQ helicase, but higher eukaryotes have more than one RecQ helicase. In human cells, the RecQ helicase family comprises five DNA helicases: RECQL1 (also known as RECQL or RECQ1), Bloom syndrome (BLM), Werner syndrome (WRN), Rothmund–Thomson syndrome (RTS; also known as RECQL4) and RECQL5 (Furuichi, 2001; Hickson, 2003; Shimamoto et al., 2004). These RecQ helicases are expressed in the nucleus, participate in DNA repair during cell proliferation and are up-regulated at the time of DNA replication (Kawabe et al., 2000; Brosh, 2013). BLM, WRN, and RTS syndromes are recessive genetic disorders of humans caused by loss of function by BLM (Ellis et al., 1995), WRN (Yu et al., 1996), and RTS helicases (Kitao et al., 1998, 1999, 2002; Lindor et al., 2000), respectively, and are characterized by genomic instability in patient cells, predisposition to various cancers and accelerated onset of aging phenotypes. RECQL1 and RECQL5 helicases, however, have no association with mutations of human disorders.

The biochemical function of DNA helicase is to unwind double-stranded DNA to single-stranded DNAs, responding to cellular DNA metabolisms, such as DNA replication and repair of DNA damage (Sharma et al., 2006; Bernstein et al., 2010). Cells in the process of replication have various endogenous DNA damage in the chromosomes resulting from base-mismatch, deletion, DNA double-strand break (DSB) or from replication errors caused by stalled replication forks and other anomalous DNA structures, such as G-quadruplex (G4) DNA. DNA damage also includes damages generated exogenously by environmental mutagens, such as ionic radiation, reactive oxygen, and genotoxic compounds. Thus, the process of DNA replication associates with many errors that expose growing cells to a risk of acquiring mutations. In proliferating cells, however, cellular DNA damage checkpoints coordinate an arrest at G1, intra-S, and G2 of the cell-cycle to allow the DNA repair process to eventually avoid mitotic catastrophe or mitosis of cells having unrepaired DNA damage. Thus, the cellular systems responsible for genome stabilization, such as DNA repair pathways and cell-cycle checkpoint functions, are indispensable for survival and proliferation of cells (Zhou and Elledge, 2000; Nyberg et al., 2002; Helleday et al., 2008). The DNA repair system contains various reactions, such as nucleotide excision repair, base excision repair, homologous recombination (HR), and non-homologous end joining (NHEJ) recombination pathways, mismatch repair pathways, and telomere maintenance for genomic integrity, in all of which RecQ helicases are considered to participate. Each member of the RecQ helicase family may achieve specific tasks by interaction with DNA repair pathway proteins (Hickson, 2003; Sharma et al., 2006) to cope with various types of DNA damage. Perhaps, accumulation of DNA damages is the most serious problem for cells because it causes cell mutagenesis, cell lethality, carcinogenesis, aging, and various other disorders. In this context, studies with retrovirus integration into host chromosomes and computational simulation have clearly shown that a single unrepaired DSB is sufficient to kill a cell deficient in DNA repair (Daniel et al., 2001). Regulation of the cell cycle and genomic integrity has also an important role in preventing carcinogenesis and in acquiring chemo-resistant malignancy in cancer cells; consequently, inhibition of these pathways in cancer cells provide a viable approach to manage cancer therapy (Helleday et al., 2008; Kelly and Fishel, 2008).

Among cellular DNA damage-responding elements, RecQ helicases directly participate in a specific DNA repair pathway and have important roles in DNA damage signaling (Frei and Gasser, 2000; Opresko et al., 2004; Pichierri et al., 2011). Based on the clarified roles of DNA helicases in the pathway of DNA repair in cancer cells, a number of strategies are suggested to fight against cancers by modulation of helicase activity (Gupta and Brosh, 2008; Brosh, 2013).

In this review, we pay special attention to recent studies on RECQL1-siRNA and WRN-siRNA that down-regulate the expression of RECQL1 and WRN helicases, respectively, and exert a tumor-specific killing effect on a wide range of tumor cells.

## INCREASED EXPRESSION OF RecQ HELICASES IN CANCER CELLS

The expression of all RecQ helicases, except RECQL5, increases when resting B cells of humans are transformed to rapidly growing B-lymphoblastoid cell line (LCL) cells by Epstein–Barr virus (EBV) infection (**Table 1**). This high expression of RecQ helicases seems to be coordinated with an increased growth rate or with augmented malignancy of tumor cells (Kawabe et al., 2000), and provides a basis for searching specific inhibitors of individual RecQ helicases. The copy number of RECQL1 and WRN helicases increases nearly 10-fold when B-LCLs are transformed from a resting state to a rapidly growing state by EBV infection, and then it is further increased several-fold after the cells are transformed to an immortalized cell state in association with a simultaneous expression of telomerase activity (**Table 1**). This continuous up-regulation of RecQ protein expression in malignant B-LCLs (up to nearly 100-fold) may reflect the need of an increased DNA damage response to resolve replicative lesions that arise in highly proliferative cell states, which may occur in other cellular systems as a consequence of oncogene-activated growth signals. Indeed, Slupianek et al. (2011) showed that BCR-ABL induces WRN mRNA and protein expression by c-MYC-mediated activation of transcription in chronic myeloid leukemia cells. Alternatively, the increased expression of DNA helicase may overcome the impaired checkpoint function of cancer cells that results from inactivation of relevant proteins by mutation or are down-regulated by selection of cells in favor of proliferation. Accordingly, inhibitors of helicase activity or RNA interference (RNAi)-mediated acute depletion of helicase should act as an intervention to increase replication lesions and also leave DNA damage unrepaired to selectively kill cancer cells. In fact, both specific small molecular weight inhibitors of RecQ helicases (Aggarwal et al., 2011, 2013; Rosenthal et al., 2013; Shadrick et al., 2013) and RNAi-mediated gene silencing with siRNA suppress the growth of cancer cells in various model systems. In the following sections, we introduce both cases, while placing a greater emphasis on the recently established RNAi-mediated strategy that proved to act specifically on cancer cells that contain impaired checkpoint functions and represent high expressions of RecQ helicases (Futami et al., 2008a,c, 2010, Arai et al., 2011; Mendoza-Maldonado et al., 2011, Sanada et al., 2013; Tao et al., 2014).

**Table 1:** Copy number of RecQ helicases in resting B cells, EBV-transformed LCL cells and telomerase-positive immortal LCL cells.

Cells	WRN	BLM	RTS	RECQL1	RECQL5
Resting B cells (N0008R)	1,200	330	120	2,100	340
Pre-immortal LCL cells (N0008T)	94,000	27,000	1,100	15,000	330
Post-immortal LCL cells (N68031M)	200,000	33,000	1,000	21,000	330

*The copy number of each RecQ helicase was determined by quantitative immunoblotting versus standard recombinant RecQ helicases. Each numeral represents a mean of duplicated samples. Reprinted from the paper by Kawabe et al. (2000)*.

## MITOTIC CATASTROPHE IN CANCER CELLS INDUCED BY RNAi-MEDIATED SILENCING OF RecQ HELICASES

**FIGURE 1:**
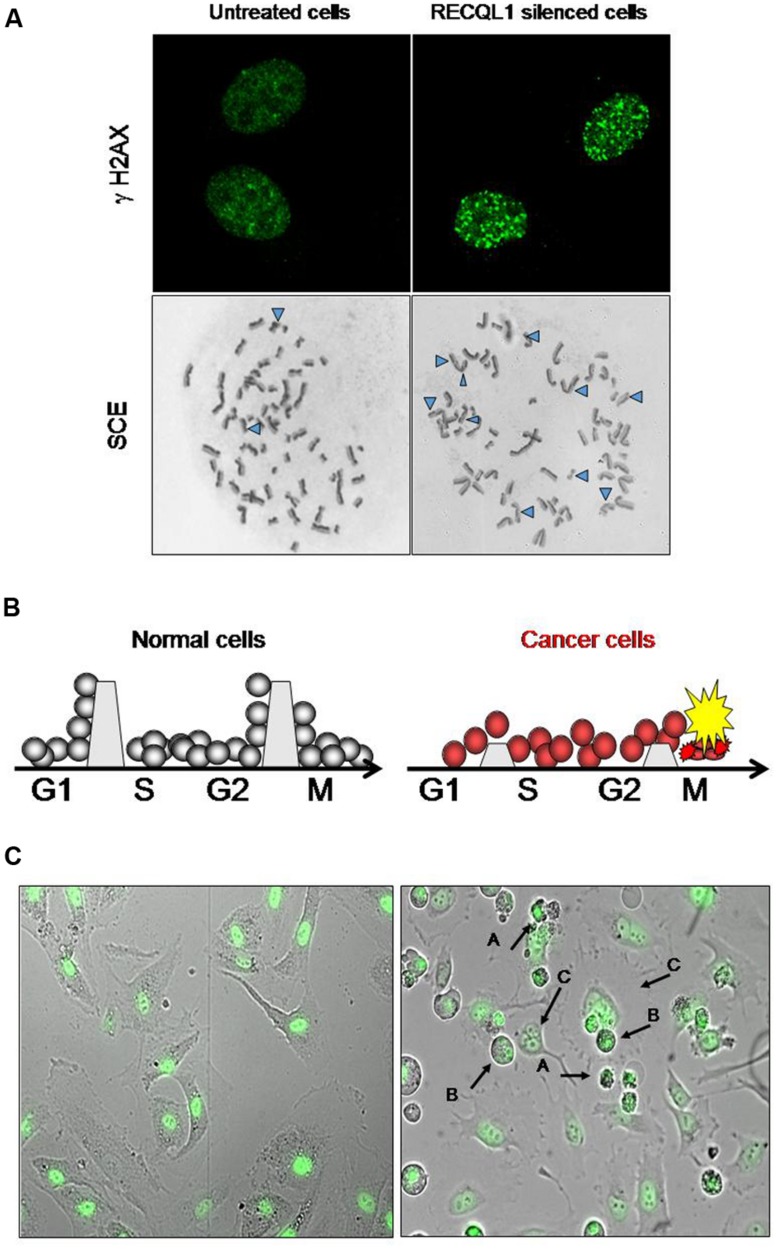
**Increase in DNA damage in cancer cells by RECQL1 silencing. (A)** Accumulation of DNA damage in RECQL1-siRNA treated cells. Control siRNA(GL3-siRNA) treated HeLa cells (left panel) and RECQL1-silenced HeLa cells (right panel) are shown. γH2AX, Detection of DSB by staining with γH2AX antibody; SCE, sister chromatid exchange regions shown by Δ. **(B)** Schematic representation of the mechanism behind cancer cell-specific mitotic death by RECQL1 silencing with RECQL1-siRNA. The boundaries set between the stage of cell cycles, which are high in normal cells and low in cancer cells, represent an image of the potency of checkpoint ability. G1, S, G2, and M are the stages of the cell cycle. **(C)** Cancer cell-specific mitotic cell death induced by RECQL1-siRNA. RECQL1 silencing *in vitro* was done in normal cells (ARPE19 cells, left panel) and cancer cells ( HeLa cells, right panel) by treatment with RECQL1-siRNA. Arrow heads show abnormal chromosomes induced by RECQL1-siRNA transfection. In the right panel **(A–C)** indicate the cells that died by mitotic catastrophe, M phase-arrested cells and multinucleated cells, respectively. Parts of the data are reprinted from the paper by Futami et al. (2008a).

In cancer chemotherapy, mitotic catastrophe is an important mechanism required to induce cell death by anticancer genotoxic agents (Roninson et al., 2001; Nitta et al., 2004). Cancer cells with an impaired G2 checkpoint function, including deficiencies in p53, p21 and 14-3-3 activities, show increased sensitivity to DNA-damaging agents, while normal cells that contain competent G2 checkpoint activity are resistant to DNA damage (Chan et al., 1999). Most cancer cells have a defective checkpoint function, and so the cell cycle of cancer cells that have DNA damage is transiently arrested at the metaphase without segregation of chromosomes, and the arrested cells subsequently undergo mitotic death. Attenuation of DNA repair capacity in cancer cells is, therefore, an effective approach to selectively kill cancer cells by using the differential capacity of checkpoint function between normal and cancer cells (**Figure 1**). Thus, inhibition of DNA repair may provide an opportunity to selectively kill cancer cells by increasing the cytotoxicity of DNA-damaging agents (and ionizing radiation) that is used for chemotherapeutic intervention. Conventional cancer chemotherapy drugs are used to induce excessive DNA damage beyond the capacity of repair in cancer cells so that cancer cells undergo cell death due to mitotic catastrophe caused by DNA damage that remains unrepaired (Nitta et al., 2004). Mitotic catastrophe occurs in replicating cells that carry DNA damage through cell cycle-mediated checkpoint regulation, and actively replicating cells are likely to be more sensitive to DNA damage than non-replicating cells (Erenpreisa and Cragg, 2001; Vogel et al., 2007). DNA damage formed endogenously during DNA replication and additional DNA damage resulting from cancer chemotherapy are all subjected to the cellular DNA repair system and are removed if the repair system is not down-regulated. Inhibitors of DNA repair pathways should augment the anticancer activity of chemotherapeutic drugs when such inhibitors are given in combination with DNA-damaging drugs. As mentioned in a later section, Futami et al. (2007) showed consistently that WRN-siRNA that silences the expression of WRN helicase and inhibits DNA repair increases the chemotherapeutic activity of camptothecin (CPT) in HeLa cells. DNA lesions that persist in the S-phase of the cell cycle inhibit progression of replication forks, resulting eventually in the formation of replication-associated DSBs of DNA (**Figure 1A**). Thus, the RecQ helicase-mediated repair mechanism must help replicating cells to remove DSB stress and prevent cells from chromosomal instability, permitting cell survival, and proliferation.

## APPLICATION OF siRNA FOR DEVELOPMENT OF INNOVATIVE ANTICANCER THERAPEUTICS

Many conventional anti-cancer chemotherapeutic drugs are small molecular weight compounds and are designed to cause DNA damage in cancer cells, including 5-FU fluorouracil nucleotides (DNA modifying antimetabolite agents), CPT derivatives (topoisomerase inhibitors), platinum derivatives (DNA crosslinking agents), Adriamycin or Doxorubicin (DNA intercalation), and cyclophosphamide (DNA alkylation), bleomycin (DNA cleavage), and mitomycin C (DNA crosslinking). Radiation therapy with either X-ray or heavy ion irradiation causes cancer cells to have DSBs by direct collision with DNA or by DNA oxidation with hydroxyl radical ions derived from irradiated water molecules. However, radiation therapy and DNA damaging chemicals used in cancer chemotherapy have drawbacks by which such treatments affect normal cells as well, often giving rise to severe cytotoxic adverse effects. Long-term chemotherapy or radiotherapy also induces cancer cells to become resistant to DNA damage by increasing the ability to repair damaged DNA (Bouwman and Jonkers, 2012).

As alternatives, siRNA drugs and micro-RNA (miRNA) drugs, which use the principle of RNAi (Novina and Sharp, 2004), have been considered as a new area of promise in anti-cancer therapy (Duxbury et al., 2004; Zhou et al., 2006). The extremely high specificity of siRNA to silence the expression of specified mRNA targets has not only been reliably applied in a wide variety of bio-medical research for gene silencing, but also has led to its emergence as a novel class of pharmaceutical drugs. RNAi is a powerful approach that can expand the anti-cancer strategy because RNAi is a natural process that is not thought to perturb normal cell physiology. Moreover, siRNA has outstanding pharmacological properties (Vaishnaw et al., 2010) by which it can catalyze the degradation of hybridized target mRNA, and, unlike other low molecular weight compounds, it is stable in the cytoplasm of transfected cells, existing in its intact structure for 1 week or more. Thus, siRNA has the properties of (i) a high specificity in binding to target mRNA, (ii) a unique catalytic profile in the mRNA degradation reaction and (iii) a stable biochemical nature in the cytoplasm that permits effective siRNA concentrations as low as nanomolar or less for more than a week, and (iv) siRNA transfection is not too harmful to cells, such that siRNA-transfected cells could be used to continue cell biological experiments. These excellent properties of siRNA predict that siRNA therapeutics would not exert serious adverse effects on cancer-peripheral normal cells.

We and others have tested candidate siRNA drugs that effectively silence the expression of RecQ helicases to attenuate cellular DNA repair activity. We studied the activity *in vitro* with cultured cancer cells and *in vivo* with human cancer-bearing xenograft animal models (Futami et al., 2007, 2008a,c, 2010; Arai et al., 2011; Mendoza-Maldonado et al., 2011; Tao et al., 2014). Recently, a first-in-man phase 1 trial was completed in the oncology field for a siRNA anti-hepatic cancer drug designed to silence two different targets of vascular endothelial growth factor-A (VEGF-A) and kinesin spindle protein (KSP) simultaneously (Tabernero et al., 2013). In that study, siRNAs were formulated with lipid nanoparticles and were administered by intravenous injection. The results indicated that the siRNA-liposome complex is tolerated in humans, is incorporated in both hepatic cells and tumor cells, and siRNA directs siRNA-sequence-matched cleavage of VEGF and KSP mRNAs in the cytoplasm of cells. This pioneering phase1 clinical study provided pharmacodynamics data that confirmed a safe siRNA-liposome complex and target mRNA-specific down-regulation in cancer cells. All these studies paved the way to make siRNA drugs truly realistic in the near future.

In the initial studies with a drug-oriented siRNA application, siRNAs were recognized to activate innate immune cells by Toll-like receptors, resulting in potent cytokine induction and immunotoxicity (Judge et al., 2005). This immune-stimulatory effect, generally associated with RNA, was thought to impair the development of RNA therapeutics. However, subsequent efforts clarified that immune recognition of siRNA is sequence-specific and is moderated by facilitating sequence design or by appropriate chemical modification of 2′-*O*-methylation at ribose moiety or both that ameliorates the immune response (Hamm et al., 2010).

## RECQL1 HELICASE AS THE TARGET OF ANTICANCER THERAPY

### BIOLOGICAL FUNCTIONS OF RECQL1 HELICASE

Of the five human RecQ helicase members, RECQL1 helicase was characterized for the first time after isolation from human cancer cells and was named RECQL1 after *E. coli* RecQ (i.e., RECQ-like human helicase number 1; Seki et al., 1994). Biochemical and cell biological data show that RECQL1 helicase unwinds DNA *in vitro* ATP-dependently, catalyzes base-matching ATP-independently (Cui et al., 2003, 2004) and resolves Holliday junctions during DNA replication in cell proliferation (Doherty et al., 2005; LeRoy et al., 2005). RECQL1 is assumed to have a role in DNA mismatch repair together with the human EXO1 and MSH2-MSH6 mismatch repair recognition complex (Doherty et al., 2005). Popuri et al. (2014) recently found that human RECQL1 participates in telomere maintenance.

RECQL1 is expressed ubiquitously in a wide variety of cells and tissues participating in maintaining the genomic integrity of cells. It is highly up-regulated in rapidly proliferating cells, particularly in carcinoma cells originating from various organs, including lung, liver, pancreas, colon, brain, ovary, and head-and-neck cancers (Futami et al., 2008a,c, 2010; Arai et al., 2011; Mendoza-Maldonado et al., 2011; Sanada et al., 2013; Tao et al., 2014). Acute depletion of human RECQL1 by RNAi renders cells sensitive to DNA damage and results in spontaneous increase in DSB-mediated gamma-H2AX foci and increased sister chromatid exchanges (SCEs), suggesting an abrogation of DNA repair (**Figure 1A**; Sharma et al., 2007; Futami et al., 2008a). Growth arrest in cancer cells by RECQL1 depletion is characterized by accumulation of unrepaired DNA damage and arrested cells at the G2 or M cell cycle phases, resulting in mitotic cell death and eventual decreased proliferation (**Figures 1A,C**).

As expected, RECQL1-silencing by RNAi technology also made cancer cells sensitive to genotoxic drugs *in vitro* (Arai et al., 2011). Mendoza-Maldonado et al. (2011) showed that human RECQL1 is highly expressed in biopsied glioblastoma tissues paralleled by a lower expression of perilesional control cells in non-dividing tissues. They showed that acute depletion of RECQL1 by RNAi results in a significant reduction of cell proliferation, perturbation of S-phase progression and an increase in spontaneous gamma-H2AX foci formation in glioblastoma cells, which become hypersensitive to anti-brain tumor drug HU or temozolomide treatment. Berti et al. (2013) showed that RECQL1 helicase has a specific role not shared with other DNA helicases in the restart of stalled replication forks induced by CPT, the reaction of which could be utilized as a new target in the search for anti-cancer drugs. Although RECQL1-deficient mice are indistinguishable from wild-type mice, their embryonic fibroblasts are sensitive to ionizing radiation (Sharma and Brosh, 2007, 2008). The function of RECQL1 helicase seems to be non-essential and complementary with other DNA repair helicases, because no human disease is known to correlate with mutations in the RECQL1 gene.

Individual variations in DNA repair capacity affects the clinical response to cytotoxic cancer therapy and overall survival of patients. Li et al. (2006a,b) and Cotton et al. (2009) found that polymorphism of A159C SNP at the 3′-untranslated region in RECQL1 genes significantly affect the overall survival of patients with resectable pancreatic cancer who were treated with adjuvant or neoadjuvant chemoradiation using gemcitabine as the radiosensor. Why the polymorphism at the non-coding region of mRNA affects the clinical progonosis of patients is not clear, but A159C SNP may function as a binding site for regulatory molecules that affect either splicing, translation efficiency, or stability of RECQL1 mRNA. In this case, the gemcitabine and radiation-induced DNA damage is likely to be repaired by the RECQL1-mediated pathway; patients with the AA genotype with A159C polymorphism showed a significantly longer survival time than those with the CC genotype, suggesting that cancer cell species having the AA genotype are sensitive to DNA damage stress, perhaps due to a reduced expression of RECQL1 helicase.

DNA repair has been termed a double-edged sword because decreased DNA repair may increase the risk of developing cancer, but, on the other hand, the decreased DNA repair of cancer cells improves survival in patients when treated with DNA-damaging agents. Generally, however, RECQL1 and other helicase genes are not mutated or are epigenetically down-regulated in tumor cells, and instead expression is most often up-regulated (Kawabe et al., 2000; Futami et al., 2008a,b,c, 2010; Sanada et al., 2013). Thus, RecQ helicases may be important survival factors for many tumors. The role of RecQ helicases in response to replication stress suggests an ideal molecular target to selectively eliminate malignant tumor cells by combined therapy with siRNA and cancer chemotherapeutic agents.

### IMMUNOHISTOCHEMICAL ANALYSIS OF RECQL1 EXPRESSIONS IN CANCERS AND ITS RELATIONSHIP TO CLINICOPATHOLOGICAL FEATURES OF CANCERS

The expression level of RECQL1 protein in hepato-cellular carcinoma (HCC) cells obtained clinically was investigated to know if the expression level of RECQL1 helicase is directly related to the differentiation stage of HCC cells, malignancy of cancer cells and prognosis of patients (Futami et al., 2010). In the relationship between RECQL1 expression and clinicopathological features, RECQL1 expression (i) significantly correlates with histological grade and MIB-1 indices of HCC, (ii) is significantly higher in simple nodular type HCC than extranodular type HCC, (iii) is significantly higher in HCC with portal vein invasion than in HCC without portal vein invasion, and (iv) is higher in HCC nodules of diameter ≥2.0 cm than in HCC nodules of diameter <2.0 cm. These data showed that RECQL1 could be used as a molecular marker to evaluate the malignancy of HCC, although RECQL1 expression and other clinicopathological features in ovary cancers do not seem to relate to prognosis, including recurrence, and survival time of patients.

In the case of ovarian cancers, an immunohistochemical study was done to test if RECQL1 expression in cancer cells can diagnose the type of cancer, such as serous type, endometrioid type, clear cell type, and mucinous type (Sanada et al., 2013). The data of that study showed that RECQL1 expression cannot distinguish type of ovarian cancer, although a trend suggests that a high expression associates with serous type and endometrical type cancers rather than with clear cell type and mucinous type cancers.

### ANTICANCER EFFECT OF RECQL1-siRNA/DDS *IN VITRO* AND *IN VIVO*

RECQL1-targeted siRNA suppresses the growth of a wide range of cultured tumor cells at low concentrations around 10 nM. Microscopic analysis showed that growth suppression is mainly due to mitotic cell death after the cell cycling is arrested at the M phase (Futami et al., 2008a, 2010). A few populations of cancer cells that survive mitotic death undergo morphological changes to non-dividing large cells that resemble the morphology of senescent cells. Notably, this same treatment with RECQL1-siRNA does not kill normal cells, such as ARPE-19, TIG3, WI38, and HUVEC cells that grow rapidly, although normal cells treated with RECQL1-siRNA slightly slow the growth rate. Thus, these experiments showed that cancer cells are extremely sensitive to depletion of RECQL1 and that cell growth is severely inhibited, as if the growth of cancer cells is dependent on a high expression of RECQL1, but non-cancerous cells and all normal cells tested are unaffected by RNAi-mediated acute depletion of RECQL1 helicase. This event was characterized as mitotic catastrophe that occurs during the cell cycle of cancer cells deficient in the checkpoint system due to accumulation of DNA damage caused by the absence of RECQL1 helicase (Futami et al., 2008a,c, 2010).

**FIGURE 2:**
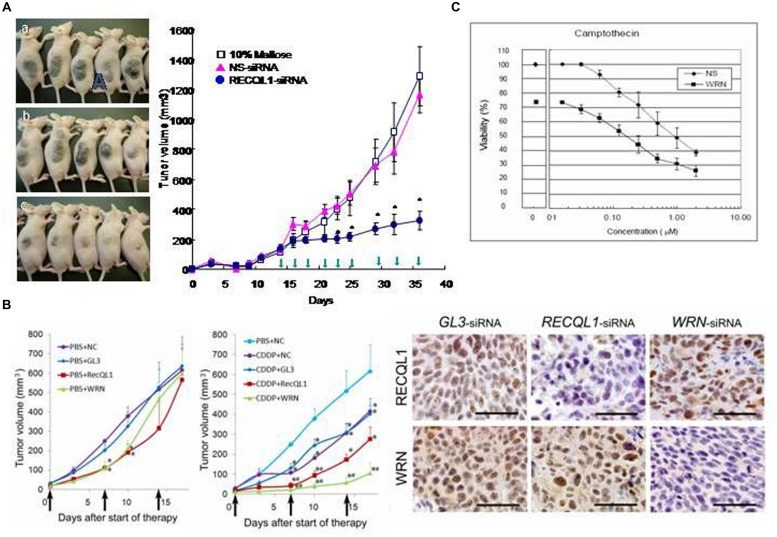
**Anticancer effects of RECQL1-siRNA and WRN-siRNA. (A)** Time course experiments measuring the inhibitory effect of the RECQL1-siRNA-LIC101 complex on the growth of Hep3B cancer nodules. Open squares, red triangles and closed circles show mice treated with 10% maltose solution as a control, NS(non-silencing)-siRNA/LIC101 complex and RECQL1-siRNA/LIC101 complex, respectively. Arrow heads represent injections made subcutaneously into cancer-bearing mice. The anticancer effect of RECQL1-siRNA/LIC101 on Hep3B cancer-bearing mice is represented visually. Panels **(A–C)** represent the size of cancer nodules grown until 42 days after inoculation. Reprinted from the paper by Futami et al. (2008b). **(B)** Anticancer effects of locally injected WRN-siRNA and RECQL1-siRNA on head and neck squamous cell carcinoma, and the synergistic effect made by the simultaneously intravenously injected genotoxic platinum drug CDDP. Therapeutic effects of WRN-siRNA and RECQL1-siRNA, and the combined effect with CDDP were examined in human FaDu carcinoma-bearing mouse xenografts. NC, no siRNA addition; GL3, GL3-siRNA; RECQL1, RECQL1-siRNA; WRN,WRN-siRNA. The right panel shows immunohistochemical evaluations of WRN and RECQL1 proteins expressed in surgically isolated cancerous regions of a human FaDu carcinoma-bearing xenograft mouse model after treatment with injections of the siRNA-atelocollagen complex. GL3-siRNA represents control siRNA that does not inhibit the expression of WRN or RECQL1 helicases. WRN-siRNA and RECQL1-siRNA treatments reduced the expression of WRN and RECQL1 proteins, respectively, stained by specific antibodies. Reprinted from the paper by Arai et al. (2011) by courtesy of those authors. **(C)** Increased cytotoxicity of anticancer drug camptothecin (CPT) in cancer cells by co-treatment with WRN-siRNA. HeLa cells were transfected with a sub-lethal concentration of WRN-siRNA and were treated with a serial dilutions of CPT added after transfection. After 48 h of culture, the cell viability was monitored. Reprinted from the paper by Futami et al. (2007). The half-lethal dose (LD 50%) of CPT was reduced by nearly ten-fold by treatment with WRN-siRNA (10 μM). Reprinted from the paper by Futami et al. (2007).

To apply this specific killing of cancer cells for clinical benefit in anticancer chemotherapy, a series of experiments were carried out with model mice inoculated with various human cancer cells and RECQL1-siRNA formulated with cationic liposome LIC-101(Yano et al., 2004). The results showed that RECQL1-targeted siRNA can suppress tumor growth in many mouse xenograft models, including lung, liver, pancreatic, colorectal cancer models and in orthotropic hepatic cancer models (**Figure 2A**), as well as in nude mice that have head and neck carcinoma (Futami et al., 2008a,c, 2010; Arai et al., 2011). Here, RECQL1-siRNA formulated with cationic liposome LIC-101 did not show any noticeable adverse effects.

In addition, RECQL1-siRNA increases the anticancer activity of *cis*-platinum (II) diamine dichloride (CDDP), a DNA-damaging cancer therapeutic, when they are administered together in a murine model of hypopharyngeal xenografts that had been inoculated with notorious FaDu cancer cells. This co-treatment of RECQL1-siRNA with CDDP increases DNA damage and cancer cell death (**Figure 2B**; Arai et al., 2011). Thus, the anticancer strategy using siRNA to attenuate the DNA repair function in cancer cells is not only superb by itself in suppressing cancer cell growth through mitotic catastrophe, but also the simultaneous treatment of genotoxic agents increases DNA damage and thus increases the efficacy of anticancer chemotherapy. Recently, Tao et al. (2014) confirmed that RECQL1 silencing by overproduction of miR-203, an miRNA that suppresses the expression of RECQL1, also leads to a strong anticancer effect on squamous carcinoma cells both *in vitro* and *in vivo*.

All these results clearly showed that RECQL1 helicase protein and RECQL1 mRNA are excellent targets for cancer chemotherapy, and that RECQL1-siRNA formulated with liposome, such as LIC-101 liposome as a drug delivery system (DDS), is a promising anticancer drug candidate. Selection of DDS, however, remains to be a key issue in the development of potential siRNA drugs, because it helps protect siRNA from degradation by ribonucleases and delivers siRNA to specified tissues, organs, and cells.

## WRN HELICASE AS THE TARGET FOR ANTICANCER THERAPY

### BIOLOGICAL FUNCTION OF WRN AS DEDUCED FROM ABERRANT DNA METABOLISM IN CELLS FROM WS PATIENTS

Werner syndrome is an autosomal recessive disease characterized by premature aging and a high incidence of malignant neoplasms. The causative gene *WRN* was identified as coding for DNA helicase belonging to the RecQ helicase family (Yu et al., 1996). The WRN protein (WRN) contains 3′->5′ exonuclease at the N-terminal region in addition to 3′->5′ DNA unwinding helicase activity, implicating its biological function in maintaining genomic stability by DNA repair. The biological function of each helicase in the RecQ family can be deduced from the outcome of aberrant DNA metabolism seen in patient cells that lack a particular RecQ helicase. In the case of WRN helicase, chromosomal instability associated with increased somatic mutation, large DNA deletions (Fukuchi et al., 1989) and unusual dynamics in telomere shortening (Tahara et al., 1997; Sugimoto, 2014) have been found in cells from WS patients, suggesting that WRN may participate in the repair of DSB through HR and NHEJ repair pathways. Because WS patients are highly predisposed to tumorigenesis, showing a particularly higher incidence of non-epithelial tumors such as soft-tissue sarcoma and benign meningioma than epithelial carcinoma that are seen frequently in normal subjects (Goto et al., 1996), we hypothesized that WRN helicase could be a tumor suppressor in normal subjects that prevents tumorigenesis of non-epithelial cells and is required for the growth of carcinoma of epithelial origin (Futami et al., 2008b). Earlier studies have clarified that most mutations in WS patients generate the premature termination codon in WRN mRNA that explains the basis of the loss of function of the *WRN* gene in WS patients (review by Shimamoto et al., 2004).

In the case of the expression of RecQ helicases, five RecQ helicase genes appear to be transcribed to mRNAs differentially depending on the organ or tissue in normal subjects. WRN seems to be expressed more or less tissue-specifically in pancreas, testis and ovary, but RECQL1 is expressed ubiquitously in all tissues (Kitao et al., 1998). Therefore, genomic instability in WS patients caused by the loss of WRN function is predicted to occur severely in tissues where WRN protein is most efficiently expressed in normal subjects. The characteristic clinical symptoms of WS patients, such as diabetes mellitus and early hypogonadism in both male and female WS patients, correspond well to organs in which WRN helicases are expressed highly in normal subjects. In those tissues or organs, DNA damage generated endogenously during DNA replication is not repaired in WS patients due to lack of the functional WRN protein. A functional relationship seems to exist between WRN and the replication checkpoint in normal cells, but an inability of this crosstalk might contribute to induce genomic instability, a common feature in senescent cells and cancer cells. In the absence of functional WRN, a series of correlated outcomes occurs, including replication arrest during cell cycling, slow growth in cell proliferation, and incomplete DNA repair resulting in many genomic deletions, all of which result in a decrease in number of cells leading to deterioration of specified tissues representing premature aging phenotypes referred to as segmental progeroid syndrome (Martin, 1985).

### ROLE OF WRN HELICASE IN TUMORIGENESIS

Transcription of the *WRN* gene is directed by a house-keeping SP1 promotor. An *in vitro* promotor analysis showed that WRN expression is attenuated by tumor suppressor p53 protein, suggesting that WRN protein, which shows a putative tumor suppressor function, decreases in p53-enriched senesced cells (Yamabe et al., 1998). In this context, Agrelo et al. (2006) showed that WRN function is abrogated in human colorectal cancer cells by transcriptional silencing associated with CpG island-promoter hypermethylation. Restoration of WRN expression by *in vitro* demethylation of promotor or by reintroduction of WRN into cancer cells induces tumor-suppressor-like features, such as reduced density of colony formation and inhibition of tumor growth, in mouse xenograft models. Analysis of many human primary tumors showed that hypermethylation of the WRN promotor is a common event in various tumor cells and is indicative of tumorigenesis by the absence of WRN expression. Agrelo et al. (2006) concluded that these findings underline the significance of WRN as a caretaker of a genome that has tumor-suppressor activity and they identify epigenetic silencing of WRN as a key step to cancer development. Decreased WRN expression may cause genomic instability in normal cells and induce initial tumorigenesis, but malignant tumor cells would acquire increased levels of WRN expression to protect their own genomic stability. Malignant tumor cell lines from various sources show a high expression of WRN helicase. In the case of colorectal cancer, the presence of aberrant hypermethylation of the WRN promotor in cancer cells predicts improved survival in patients treated with a CPT derivative irinotecan, a topoisomerase inhibitor used in therapeutics of this neoplasm, because decreased WRN makes neoplasmic cells sensitive to a DSB-inducing genotoxic agent (Agrelo et al., 2006). WS patients have a high frequency of having non-epithelial tumors than epithelial carcinoma, but the underlying reason remains to be clarified. However, a high WRN expression may possibly be a prerequisite to maintain genomic integrity in rapidly growing carcinoma cells in normal subjects.

Futami et al. (2007) showed that WRN-siRNA abrogates the DNA repair activity of HeLa cells and increases the sensitivity to topoisomerase inhibitor CPT. In this case, the silencing of WRN helicase prevents the repair of CPT-induced DNA damage and permits accumulation of unrepaired DNA damage that explains increased cytotoxicity of CPT in the combined treatment of CPT and WRN-siRNA (**Figure 2C**). The data also explained why WS patient cells also show increased sensitivity to other genotoxic reagents, including CPT, etoposide, 4NQO, and bleomycin (Sakamoto et al., 2001). Recently, Aggarwal et al. (2011) used small molecular weight compound NSC19630 that inhibits WRN activity in anti-cancer cell biology experiments. Exposure of human cancer cells to NSC 19630 dramatically impairs growth and proliferation, induces apoptosis WRN-dependently and results in increased γ-H2AX reactive chromosomal DSB, similar to the results obtained by Futami et al. (2007) by silencing WRN with siRNA treatment. NSC 19630 exposure leads to delayed S-phase progression, consistent with accumulation of stalled replication forks, and to DNA damage WRN-dependently. Exposure to NSC 19630 sensitizes cancer cells to the G4-binding compound telomestatin or to a poly(ADP-ribose) polymerase (PARP) inhibitor. Sublethal dosage of NSC 19630 and the chemotherapy drug topotecan acts synergistically to inhibit cell proliferation and induce DNA damage (Aggarwal et al., 2011). The use of this WRN helicase inhibitor (NSC 19630) may provide insight into the importance of WRN-mediated pathways important for DNA repair and the replicational stress response.

### ANTICANCER EFFECT OF WRN-siRNA/DDS *IN VITRO* AND *IN VIVO*

Both WRN and RECQL1 helicases are expressed highly in head and neck squamous carcinoma cells (HNSCCs; **Figure 2B**), and siRNA-mediated silencing of either gene suppresses growth of HNSCC *in vitro* (Arai et al., 2011). Similarly, local injections of WRN-siRNA and RECQL1-siRNA formulated with atelocollagen into a mouse zenograft model of hypopharyngeal carcinoma markedly inhibits tumor growth. A combination of either siRNA with CDDP, a genotoxic drug commonly used in HNSCC treatment, significantly augments the *in vivo* anticancer effect of CDDP (**Figure 2B**). Notably, no recurrence was observed for some tumors after siRNA and CDDP treatment in this model. These findings offer a preclinical proof of WRN and RECQL1 helicase as novel therapeutic targets to treat aggressive HNSCC and possibly other cancers.

Several lines of evidence suggest that WRN helicase has an important role in DNA replication and S-phase progression (review by Croteau et al., 2014). Loss of WRN markedly extends the time of cell cycles after genotoxic treatments. This result comfirmed the importance of WRN during genomic replication, and indicated that WRN acts to facilitate fork progression after DNA damage or replication arrest. Recent studies showed that conditional gene silencing of WRN expression in non-small-cell lung cancer xenografts that are over-expressed with c-MYC inhibits tumor growth, suggesting that targeting WRN protein inhibits growth of c-MYC-associated cancers (Moser et al., 2012). A growing body of evidence shows that WRN functions in close connection with the replication checkpoint that includes both ataxia telangiectasia mutated (ATM) protein and ATM-Rad3-related (ATR) kinase activities and maintains fork integrity and re-establishment of fork progression. Recent findings also support the view that ATR and ATM kinases modulate WRN function at defined steps of the response to replication fork arrest. Or *vice versa*, WRN may be required to activate ATM kinase and the intra-S-phase checkpoint in response to DNA interstrand crosslink-induced DSBs and other forms of fork arrest (Cheng et al., 2008; Pichierri et al., 2011). WRN is speculated to be a DNA helicase that efficiently unwinds G4 DNA and other forms of alternative DNA structures, such as telomeric displacement loops. These data indicate that DNA repair helicases, such as RECQL1 and WRN, represent a novel class of important targets required for anticancer therapy. Inhibition of their activities either by siRNA or by small molecular weight compounds in cancer cells having checkpoint mutation should give rise to a new type of anticancer therapeutic drug that also permits strong synergistic anticancer effects when combined with genotoxic drugs as depicted in **Figure 2B** (with CDDP) and **Figure 2C** (with CPT; Futami et al., 2007; Arai et al., 2011).

## BLM, RTS, AND RECQL5 HELICASES ARE POTENTIAL TARGETS OF ANTICANCER THERAPY

Patients of BLM and a subset of patients with RTS who lack functional BLM and RTS helicases, respectively, manifest clinical phenotypes of genomic instability and a high incidence of cancers (Ellis et al., 1995; Lindor et al., 2000). Like *WRN*, helicase genes *BLM* and *RTS* are suggested to function as tumor suppressor genes in normal individuals. Both BLM and RTS are expressed highly in rapidly growing EBV-infected LCL cells from normal individuals, and the expression of BLM is, like WRN, further augmented when EBV-transformed cells are differentiated to telomerase-positive immortal cells (**Table 1**). In a pioneering effort to find an antiproliferative drug, Nguyen et al. (2013) discovered small molecular weight compound ML216 from a screening of a chemical compound library that inhibits the DNA binding activity of BLM *in vitro* (IC50% ∼3 uM) and abrogates the unwinding activity of BLM on a forked DNA duplex. As expected, ML216 increased the rate of SCE in cultured human fibroblasts and increased the sensitivity to aphidicolin, an inhibitor of replicative DNA polymerases (Banerjee et al., 2013; Rosenthal et al., 2013). However, ML216 does not seem to be highly specific to BLM helicase, because it also inhibits DNA unwinding activity of WRN (Banerjee et al., 2013). Further, pharmacological studies may be needed to improve the specificity and efficacy before ML216 is tested for clinical application.

Mao et al. (2010) showed that depletion of BLM by RNAi-mediated shRNA gene silencing suppresses the growth of osteosarcoma U2OS cell line with an increase in DNA damage detected by gamma-H2AX, suggesting that BLM is a potential target for anticancer therapeutics. They also showed depletion of BLM and WRN by shRNA from U2OS and normal fibroblast sensitized cells to dose-dependent killing by CPT, CDDP and 5-FU, consistent with previous observations by Futami et al. (2007) in which WRN-siRNA treatment sensitized HeLa cells to CPT and lowered the cytotoxic dose by nearly 10-fold.

Few reports exist of RTS and RECQL5 helicases as possible cancer targets. However, Su et al. (2010) elegantly showed that RECQL4 (RTS) helicase has critical roles in prostate carcinogenesis, and that RNAi-mediated gene suppression reduces the growth and survival of metastatic prostate cancer cells. In addition, RECQL4-suppressed cells treated with siRNA show increased apoptotic death after treatment with ultraviolet C and γ-ray radiation. Thus, Su et al. (2010) conclude that RECQL4 protects the genomic integrity of prostate cancer cells from endogenous and exogenous DNA damage, similar to our discussion above with RECQL1. In fact, RECQL4-depleted prostate cancer cells undergo extensive apoptotic death in PARP -1-dependently *in vitro* and show reduced tumorigenicity in nude mice *in vivo*. Because the expression of RECQL4 increased highly in metastatic prostate cancer cells and in tumor tissue, RECQL4 protein could be used as new tumor marker.

While much insight has been gained into the function of WRN and BLM, little is known about RECQL5. Three isomers result from differential splicing from the RecQ gene transcript. The largest molecule, RECQL5, localizes to the nucleus, whereas the two smaller molecules are found in the cytoplasm (Shimamoto et al., 2000). Nuclear RECQL5 has a role in maintaining genomic stability by cooperating with topoisomerase II(alpha) in DNA decatenation and cell cycle progression (Ramamoorthy et al., 2012). Because RECQL5- depleted cells show slow proliferation, G2 and M cell cycle arrests and late S-phase cycling defects, resulting in entangled abnormal chromosomes at the M phase, possibly RECQL5 could be challenged, like RECQL1, as a new cancer therapeutic target.

## PERSPECTIVE

The relation between helicases and DNA damage response and genomic stability suggests that DNA repair helicases may be suitable targets for cancer chemotherapy (Helleday et al., 2008). Most cancer cells are deficient in G1 and G2 checkpoint function and fail to arrest the cell cycle at G1 and G2 phases to engage in DNA repair. Instead, cells that proceed in the cell cycle to the M phase, where DNA repair is no longer permitted, eventually undergo cell death as they enter mitosis (Nitta et al., 2004). Several studies have reported a greater efficacy in the therapeutic application of siRNA against malignant tumors in combination with other chemotherapeutic agents, such as with gemcitabine together with ribonucleotide reductase-siRNA (Duxbury et al., 2004), CPT together with WRN helicase-siRNA (Futami et al., 2007) and Adriamycin together with survivin-siRNA (Yonesaka et al., 2006). Such approaches of targeting RecQ helicases with a strict specificity, such as by gene silencing with siRNA, would be most conspicuous in cooperation with DNA damaging agents or radiation. Thus, DNA repair helicases expressed highly in most malignant and rapidly proliferating cancer cells not only provide new proliferative protein markers, such as WRN and RECQL1 proteins (**Figure 2**), but also provide logical consequences for combination therapy to fight against drug-resistant cancer cells.

In conclusion, DNA repair helicases are ideal molecular targets for anticancer drug discovery, and, in particular, RECQL1-siRNA and WRN-siRNA and perhaps BLM-siRNA and RTS-siRNA are promising new therapeutic interventions against malignant cancers of various histological and clinical characteristics, including, for example, those of the platinum-resistant, CPT-resistant, and 5-FU-resistant subtypes.

## Conflict of Interest Statement

The authors declare that the research was conducted in the absence of any commercial or financial relationships that could be construed as a potential conflict of interest.
